# A multivariable prediction model combining 18F-PSMA PET/CT and mpMRI for clinically significant prostate cancer: development and validation

**DOI:** 10.3389/fonc.2026.1835850

**Published:** 2026-05-18

**Authors:** Chaojian Yu, Zihou Zhao, Peidong Tian, Jingcheng Zhou, Lin Cai, Jianhui Qiu, Kan Gong

**Affiliations:** 1 Department of Urology, Peking University First Hospital, Beijing, China; 2 Institute of Urology, Peking University, Beijing, China; 3 National Urological Cancer Center, Beijing, China

**Keywords:** clinically significant prostate cancer, multiparametric MRI, prediction model, prostate cancer, PSMA PET/CT

## Abstract

**Background:**

Multiparametric MRI (mpMRI) and prostate-specific membrane antigen (PSMA) PET/CT provide complementary information for prostate cancer diagnosis, but optimal integration strategies remain unclear. We aimed to develop and validate a multivariable model combining clinical and imaging parameters to predict clinically significant prostate cancer (csPCa).

**Methods:**

This study included 1305 consecutive patients with suspected prostate cancer who underwent both mpMRI and 18F-PSMA PET/CT with biopsy at Peking University First Hospital. The development and temporal validation cohorts were temporally divided; the development cohort was split into training (70%) and internal test (30%) sets. csPCa was defined as Gleason Grade Group ≥2. Predictors were selected using LASSO regression (1-standard error criterion) followed by forward stepwise logistic regression (AIC, *p* < 0.05). Model performance was assessed by AUC, calibration plots, and decision curve analysis.

**Results:**

The final model retained 3 predictors: PRIMARY score, PI-RADS score, and PSAD. The training AUC was 0.916. At the Youden Index cutoff (≥84%), sensitivity was 79.3% and specificity 76.6%; at the recommended screening cutoff (≥46%), sensitivity reached 96.0%. The AUC was 0.914 (95% CI: 0.882–0.941) in the internal test set and 0.837 (95% CI: 0.778–0.891) in temporal validation. The model showed good calibration (Brier score: 0.096) and superior clinical utility over individual imaging parameters.

**Conclusions:**

The multivariable model integrating 18F-PSMA PET/CT and mpMRI parameters provides accurate risk stratification for csPCa and may help optimize biopsy decisions.

## Introduction

Prostate cancer (PCa) is the most commonly diagnosed malignancy in men worldwide (1). While early detection through prostate-specific antigen (PSA) screening has increased the diagnosis of localized disease, it has also led to substantial overdiagnosis and overtreatment of indolent cancers that would never cause clinical harm (2). This has driven a paradigm shift toward identifying clinically significant prostate cancer (csPCa), which requires active treatment, while avoiding unnecessary interventions for low-risk disease. However, the definition of csPCa has varied across studies, creating challenges for clinical decision-making and research comparisons. Based on the 2014 International Society of Urological Pathology (ISUP) consensus conference and subsequent clinical guidelines, csPCa is now widely defined as Gleason Grade Group (GG) ≥2 (Gleason score ≥3 + 4), a threshold that identifies tumors with metastatic potential warranting definitive treatment (3).

Traditional screening with prostate-specific antigen (PSA) testing has been limited by its inability to distinguish csPCa from indolent disease, leading to overdiagnosis and overtreatment (4). Multiparametric magnetic resonance imaging (mpMRI) has emerged as a valuable tool for pre-biopsy risk stratification. The Prostate Imaging Reporting and Data System (PI-RADS) provides a standardized framework for lesion assessment, with PI-RADS ≥3 triggering targeted biopsy consideration (5). Landmark trials including PROMIS and PRECISION have established mpMRI as a gatekeeper for prostate biopsy (6, 7). However, mpMRI has notable limitations, including moderate interobserver variability and suboptimal specificity, particularly for PI-RADS 3 lesions (8). PSA density (PSAD), calculated as serum PSA divided by prostate volume, has also been shown to improve diagnostic specificity (9).

Prostate-specific membrane antigen (PSMA) positron emission tomography/computed tomography (PET/CT) has gained attention as a molecular imaging modality with high tumor specificity. PSMA is a type II transmembrane glycoprotein overexpressed in prostate cancer cells, making it an ideal target for molecular imaging (10). While 68Ga-labeled PSMA tracers were initially developed, 18F-labeled PSMA ligands offer several advantages including longer half-life, higher image resolution, and cyclotron-based production enabling wider availability (11). Our institution has developed 18F-PSMA-137, an 18F-labeled PSMA ligand with favorable pharmacokinetic properties and reduced urinary excretion, improving pelvic visualization (12). The PRIMARY scoring system provides a standardized qualitative assessment framework for intraprostatic PSMA uptake (13).

The integration of mpMRI and PSMA PET may provide complementary diagnostic information, combining anatomical detail with molecular characteristics. However, the optimal method for combining these modalities remains unclear. Simple rule-based approaches may sacrifice specificity, while requiring concordant positive findings may miss cancers.

Therefore, this study aimed to develop and validate a multivariable prediction model integrating clinical parameters, mpMRI findings, and 18F-PSMA PET/CT data to improve pre-biopsy risk stratification for csPCa.

## Materials and methods

### Study design and population

This diagnostic accuracy study employed a combined retrospective-prospective design with consecutive patient enrollment. Male patients aged ≥18 years with suspected PCa who underwent both mpMRI and 18F-PSMA PET/CT with prostate biopsy at Peking University First Hospital were enrolled retrospectively from August 2021 to August 2025 and prospectively from September 2025 to December 2025. All patients meeting the inclusion criteria during the respective time periods were consecutively included to minimize selection bias.

The indications for biopsy included: (1) suspicious nodules on digital rectal examination (DRE); (2) elevated serum PSA level (>4 ng/mL); or (3) suspicious lesions identified on imaging.

Patients were excluded based on the following criteria: (1) time interval exceeding 3 months between imaging modalities and biopsy; (2) history of prostate surgery, including transurethral resection of the prostate (TURP); (3) prior treatment for PCa, including androgen deprivation therapy (ADT), radiotherapy, or focal therapy; (4) incomplete imaging or pathological data.

The study was approved by the Institutional Ethics Committee (Approval No. 2025R0380-0002), with waiver of informed consent for the retrospective cohort and written informed consent obtained from all participants in the prospective cohort.

### Clinical and pathological data

Baseline clinical characteristics including age at the time of biopsy, serum PSA level (ng/mL), and prostate volume (PV, in mL) measured on imaging were collected. PSA density (PSAD) was calculated as PSA/PV (9). The reference standard was histopathological diagnosis from systematic (9 or 12-core) and/or MRI-targeted prostate biopsy. The reference standard for all patients was histopathological diagnosis via a prostate biopsy. Biopsy results were graded per ISUP 2014 consensus (3). The primary endpoint of the study was csPCa defined as ISUP GG ≥ 2 (Gleason score ≥ 3 + 4 = 7). The patients who had GG 1 (Gleason score of 3 + 3 = 6) or benign pathology were deemed not to have csPCa.

### Image acquisition and interpretation

All mpMRI examinations were performed on 1.5T or 3.0T scanners, with T2-weighted imaging (T2WI) and diffusion-weighted imaging (DWI) sequences acquired according to PI-RADS v2.1 guidelines (5). 18F-PSMA PET/CT was performed 60 minutes after intravenous injection of 18F-PSMA-137. All images were reviewed by two independent readers (board-certified radiologists with over 10 years of experience in prostate MRI, and nuclear medicine physicians with over 10 years of experience in PSMA PET/CT) blinded to pathology results.

For mpMRI, the PI-RADS v2.1 scoring system was applied (28). T stage was determined based on MRI findings according to the TNM classification criteria, assessing for prostate-confined lesion (T2), extracapsular extension (T3a), seminal vesicle invasion (T3b), and invasion of adjacent structures (T4). It should be noted that this MRI-based T staging differs from clinical T staging defined in international guidelines, which is based on DRE findings; MRI-based staging was used in this study as it provides more detailed anatomical assessment for pre-biopsy risk stratification.

For 18F-PSMA PET/CT, the PRIMARY scoring system was used for qualitative assessment (detailed scoring criteria provided in **Supplementary Table 1**) (13). Briefly, a PRIMARY score using a combination of pattern information and SUVmax was assigned to each patient: Score 1 indicated no pattern and low-grade activity; Score 2 indicated diffuse transition zone (TZ) or symmetric central zone (CZ) activity without focal uptake; Score 3 indicated focal TZ activity visually greater than twice the background TZ activity; Score 4 indicated focal peripheral zone (PZ) activity; Score 5 indicated any pattern with SUVmax ≥12. The maximum standardized uptake value (SUVmax) of the dominant lesion was also recorded for quantitative analysis. Discrepancies were resolved by consensus.

### Data splitting strategy

The study utilized a internal-temporal validation framework. The development cohort and temporal validation cohort were temporally divided with February 2025 as the cutoff point. The development cohort was randomly split using stratified sampling (maintaining outcome proportions) into: (1) a training set (70%) for model construction and coefficient estimation, and (2) an internal test set (30%) for initial performance evaluation on unseen data from the same time period. The temporally separated temporal validation cohort served as independent validation to assess model generalizability across different time periods.

This design allows assessment of: (1) reproducibility on contemporaneous unseen data in the internal test set, and (2) temporal transportability in the temporal validation cohort.

### Statistical analysis

Continuous variables were expressed as medians with interquartile ranges (IQR) and compared using Mann-Whitney U test. Categorical variables were reported as frequencies with percentages and compared using chi-square test.

Continuous variables, including age (cutoff 60 years), PSAD (cutoff 0.15 ng/mL2), and SUVmax (cutoffs 4 and 8), were categorized based on clinically meaningful thresholds (14). To address potential computational collinearity, PSAD was included as a candidate variable while PSA and prostate volume were excluded (9). Candidate variables from the training set were entered into least absolute shrinkage and selection operator (LASSO) regression for initial variable selection (15), with the optimal regularization parameter (λ) determined by 10-fold cross-validation using the 1-standard error (1se) criterion. The selected variables were then forwarded to a stepwise logistic regression model based on the Akaike Information Criterion (AIC) and a multivariable significance threshold of *p* < 0.05 for final model construction. The optimal cutoff of the model was determined by calculating the Youden Index, while a recommended screening threshold was also identified for optimal clinical prioritization. At these two specific thresholds, the sensitivity, specificity, positive predictive value (PPV), and negative predictive value (NPV) were calculated.

Model assessment included internal testing and temporal validation. To address potential overfitting and assess model stability, internal validation was also performed using 1000 bootstrap resamples to calculate the optimism-corrected area under the curve (AUC). The probabilities calculated by the models were used to draw receiver operating characteristic (ROC) curves (16). Model discrimination was evaluated using the area under the curve (AUC) with 95% confidence intervals (CI). Model calibration was assessed using calibration plots and the Brier score (17). Decision curve analysis (DCA) evaluated clinical utility across a range of threshold probabilities (18).

**Figure 1:**
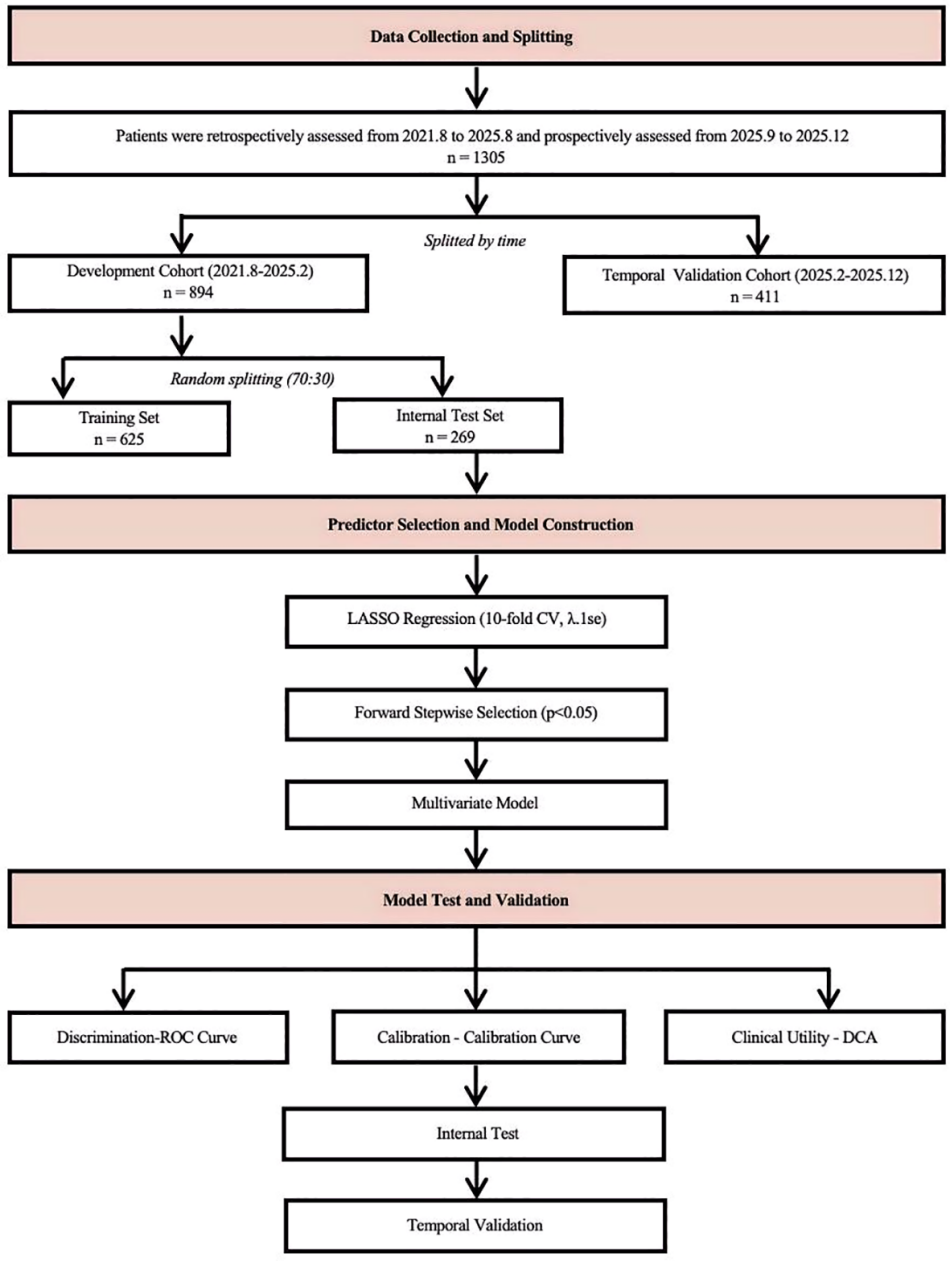
The study flowchart. CV, cross validation; ROC, receiver operating characteristic; DCA, decision curve analysis.

The study flowchart was shown in **Figure 1**. Statistical analyses were performed using Python 3.9 with scikit-learn and statsmodels packages (19). Two-sided *p* < 0.05 was considered statistically significant.

## Results

### Patient characteristics

A total of 1305 consecutive patients were included: 894 in the development cohort (training set: n=625; internal test set: n=269) and 411 in the temporal validation cohort. The baseline characteristics are summarized in **Table 1**.

**Table 1:** Baseline patient characteristics (development and validation cohorts).

Characteristic	Development (n=894)	Validation (n=411)	*p*-value
Age, year	68.0 [62.0-73.0]	69.0 [63.0-73.0]	0.129
PSA, ng/mL	10.1 [6.9-14.0]	10.6 [7.5-16.0]	<0.001
PV, cmL	40.5 [31.0-57.5]	36.6 [27.4-49.1]	<0.001
PSAD, ng/mL^2^	0.2 [0.1-0.3]	0.3 [0.2-0.5]	<0.001
SUVmax	9.6 [5.5-17.6]	10.3 [5.7-17.7]	0.198
T stage			<0.001
≤ T2	605 (67.7%)	310 (75.4%)	
T3a	152 (17.0%)	36 (8.8%)	
T3b	119 (13.3%)	51 (12.4%)	
T4	18 (2.0%)	14 (3.4%)	
PI-RADS Score			0.182
1-2	79 (8.8%)	30 (7.3%)	
3	153 (17.1%)	56 (13.6%)	
4	352 (39.4%)	162 (39.4%)	
5	310 (34.7%)	163 (39.7%)	
PRIMARY Score			0.082
1-2	166 (18.6%)	54 (13.1%)	
3	83 (9.3%)	46 (11.2%)	
4	275 (30.8%)	136 (33.1%)	
5	370 (41.4%)	175 (42.6%)	
Biopsy			
Total cores	11.0 ± 3.0	10.4 ± 3.0	<0.001
Positive cores	4.4 ± 2.9	5.2 ± 2.8	<0.001
Positive rate, %	42.69 ± 28.72	53.07 ± 29.0	<0.001
ISUP Grade Group			0.003
Benign	112 (12.5%)	28 (6.8%)	
1	82 (9.2%)	36 (8.8%)	
2	285 (31.9%)	152 (37.0%)	
3	201 (22.5%)	103 (25.1%)	
4	88 (9.8%)	52 (12.7%)	
5	126 (14.1%)	40 (9.7%)	
csPCa (ISUP≥2)	700 (78.3%)	347 (84.4%)	0.012

PSA, prostate specific antigen; PV, prostate volume; PSAD, PSA density; SUVmax, maximum standardized uptake value; PI-RADS, Prostate Imaging Reporting and Data System; ISUP, International Society of Urological Pathology; csPCa, clinically significant prostate cancer.

Continuous variables are presented as median [IQR]. Categorical variables are presented as count (%). P values from Mann-Whitney U test (continuous) or chi-square test (categorical).

csPCa was detected in 700 patients (78.3%) in the development cohort and 347 patients (84.4%) in the temporal validation cohort. The temporal validation cohort showed higher median PSA, PSAD, and SUVmax compared to the development cohort (all *p* < 0.05), reflecting temporal changes in referral patterns.

### Predictor selection

**Figure 2:**
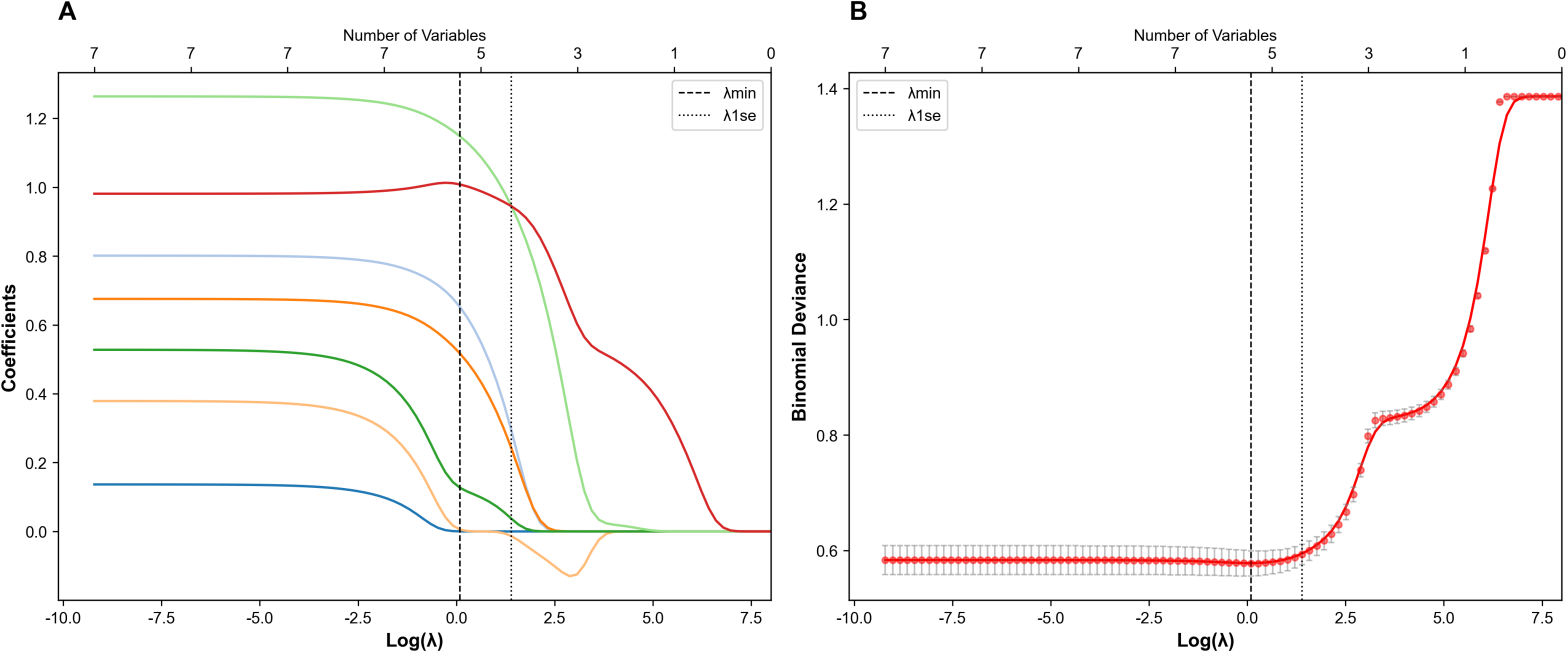
Variable selection by LASSO regression analysis. **(A)** LASSO Regression Convergence Path Plot. **(B)** 10-Fold Cross-Validation Plot.

**Figure 3:**
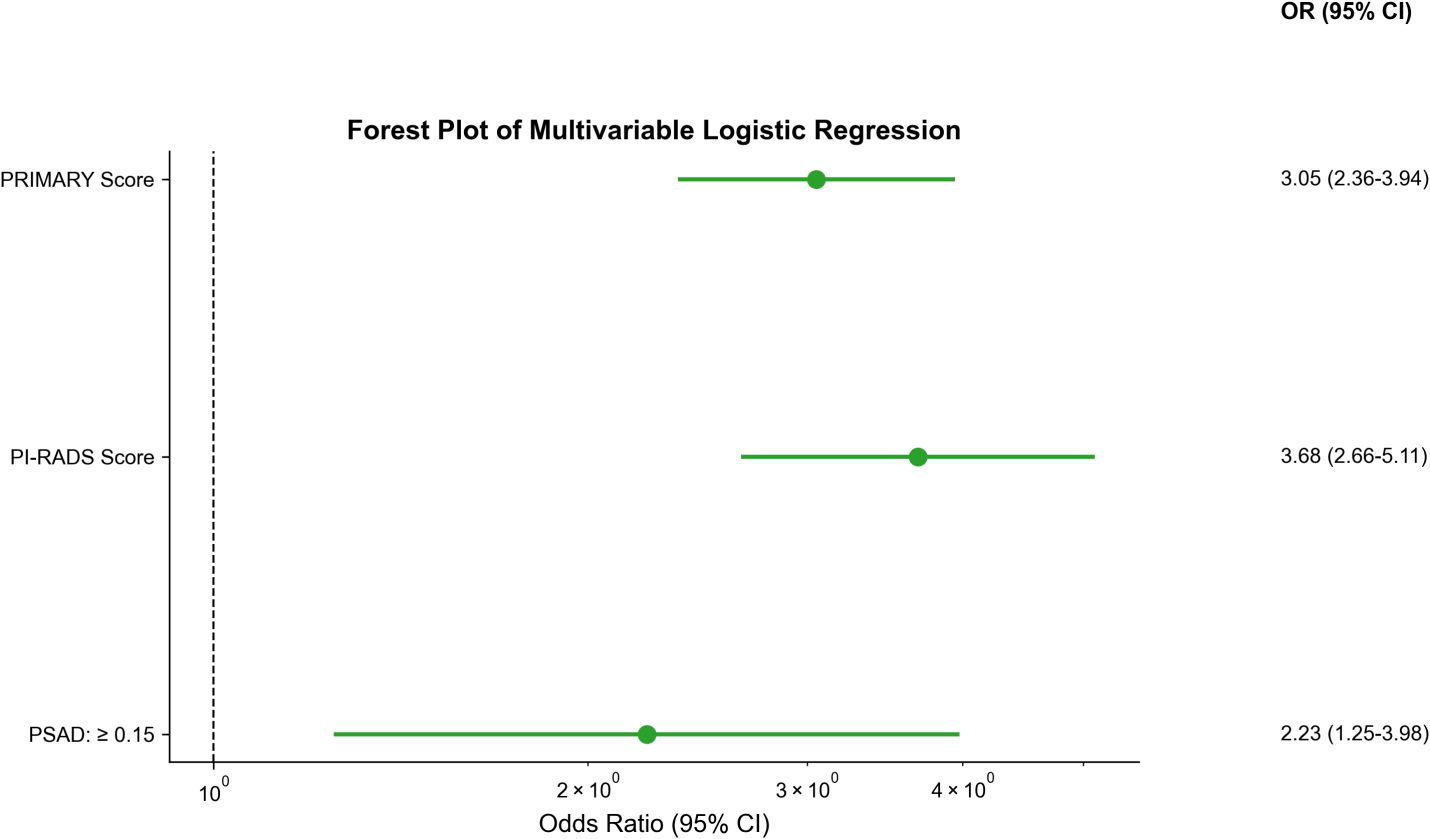
Forest plot for predicting the presence of csPCa. PSAD, prostate specific antigen density; PI-RADS, Prostate Imaging Reporting and Data System.

Candidate variables from the training set (**Table 2**) were entered into LASSO regression. LASSO regression with 10-fold cross-validation using the 1se criterion identified 5 macro-variable groups: PSAD, MRI-based T stage, SUVmax, PI-RADS score, and PRIMARY score (**Figure 2**). These candidate variables were then subjected to forward stepwise regression based on AIC and multivariable *p* < 0.05. The stepwise process refined the predictors, ultimately retaining three core variables: PSAD, PI-RADS score, and PRIMARY score. (**Table 3**, **Figure 3**).

**Table 2:** Candidate variables characteristics of training set.

Variable	csPCa (+) (n=489)	csPCa (−) (n=136)	*p*-value
Age, year	68.0 [63.0-73.0]	66.0 [61.0-72.0]	0.010
< 60	62 (12.7%)	29 (21.3%)	0.017
≥ 60	427 (87.3%)	107 (78.7%)	
PSAD, ng/mL²	0.2 [0.2-0.4]	0.1 [0.1-0.2]	<0.001
< 0.15	87 (17.8%)	61 (44.9%)	<0.001
≥ 0.15	402 (82.2%)	75 (55.1%)	
T stage			<0.001
≤ T2	294 (60.1%)	129 (94.9%)	
> T2	195 (39.9%)	7 (5.1%)	
SUVmax	11.6 [6.6-19.6]	4.8 [4.0-6.2]	<0.001
< 4	17 (3.5%)	33 (24.3%)	<0.001
4-8	142 (29.0%)	81 (59.6%)	
≥ 8	330 (67.5%)	22 (16.2%)	
PI-RADS Score			<0.001
1-2	8 (1.6%)	47 (34.6%)	
3	63 (12.9%)	47 (34.6%)	
4	215 (44.0%)	35 (25.7%)	
5	203 (41.5%)	7 (5.1%)	
PRIMARY Score			<0.001
1-2	24 (4.9%)	90 (66.2%)	
3	49 (10.0%)	7 (5.1%)	
4	176 (36.0%)	27 (19.9%)	
5	240 (49.1%)	12 (8.8%)	

PSA, prostate specific antigen; PV, prostate volume; PSAD, PSA density; SUVmax, maximum standardized uptake value; PI-RADS, Prostate Imaging Reporting and Data System; ISUP, International Society of Urological Pathology; csPCa, clinically significant prostate cancer.

Continuous variables are presented as median [IQR]. Categorical variables are presented as count (%). P values from Mann-Whitney U test (continuous) or chi-square test (categorical).

**Table 3:** Variables of multivariable logistic regression model.

Variable	OR	95% CI	*p*-value
PRIMARY Score	3.05	2.36-3.94	<0.001
PI-RADS Score	3.68	2.66-5.11	<0.001
PSAD			0.007
< 0.15	Reference		
≥ 0.15	2.23	1.25-3.98	0.007

OR, odds ratio; CI, confidence interval; PSAD, prostate specific antigen density; PI-RADS, Prostate Imaging Reporting and Data System.

Forward stepwise regression analysis was used to select variables.

Among the retained imaging parameters, PI-RADS score showed the strongest association with csPCa (OR = 3.68, 95% CI: 2.66-5.11, *p* < 0.001). PRIMARY score also demonstrated strong independent predictive value (OR = 3.05, 95% CI: 2.36-3.94, *p* < 0.001). Elevated PSAD (≥0.15 ng/mL2) was a significantly independent clinical predictor (OR = 2.23, 95% CI: 1.25-3.98, *p* = 0.007).

### Model development and performance

**Figure 4:**
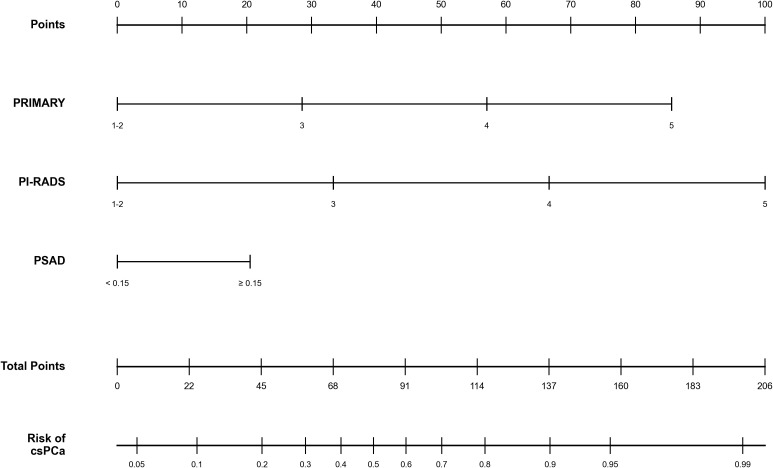
Nomogram for predicting the presence of csPCa. PSAD, prostate specific antigen density; PI-RADS, Prostate Imaging Reporting and Data System.

**Figure 5:**
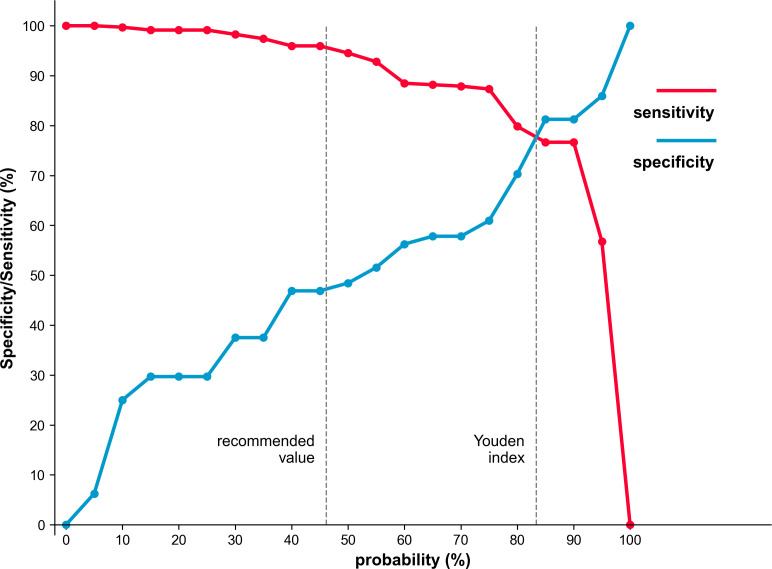
Sensitivities and specificities of the developed nomograms for predicting csPCa.

The final multivariable model strictly included three predictors: PSAD, PI-RADS score, and PRIMARY score. A point-based nomogram incorporating the three selected predictors was constructed to facilitate clinical application (**Figure 4**). In this nomogram, each predictor is assigned a specific point value on the top axis, and the sum of these points maps directly to the estimated probability of csPCa on the bottom axis (20). The optimal threshold determined by the Youden index was ≥84% probability, which yielded a sensitivity of 79.3% and specificity of 76.6%. A clinically recommended threshold of ≥46% probability was also provided to maximize csPCa detection, achieving a sensitivity of 96.0% and specificity of 46.6% (**Figure 5**, **Table 4**).

**Table 4:** Clinical Application cut-offs values and model diagnostic performance.

Cut-off values of predictive probabilities	Sensitivity (ratio) 95% CI	Specificty (ratio) 95% CI	PPV (ratio) 95% CI	NPV (ratio) 95% CI
Youden index≥84%	79.3% (275/347) 74.7%-83.2%	76.6% (49/64) 64.9%-85.3%	94.8% (275/290) 91.6%-96.8%	40.5% (49/121) 32.2%-49.4%
recommended value≥46%	96.0% (333/347) 93.3%-97.6%	46.9% (30/64) 35.2%-58.9%	90.7% (333/367) 87.3%-93.3%	68.2% (30/44) 53.4%-80.0%

CI, confidence interval; PPV, positive predictive value; NPV, negative predictive value.

The Youden index cut-off values of predicting probabilities were obtained at the maximum Youden index (sensitivity + specificity − 1).

We recommended a cut-off value of 46% for the csPCa-predicting nomogram to achieve the sensitivity of 96.0%.

The nomogram allows calculation of individualized probability of csPCa based on patient-specific clinical and imaging characteristics.

### Model test and validation

**Figure 6:**
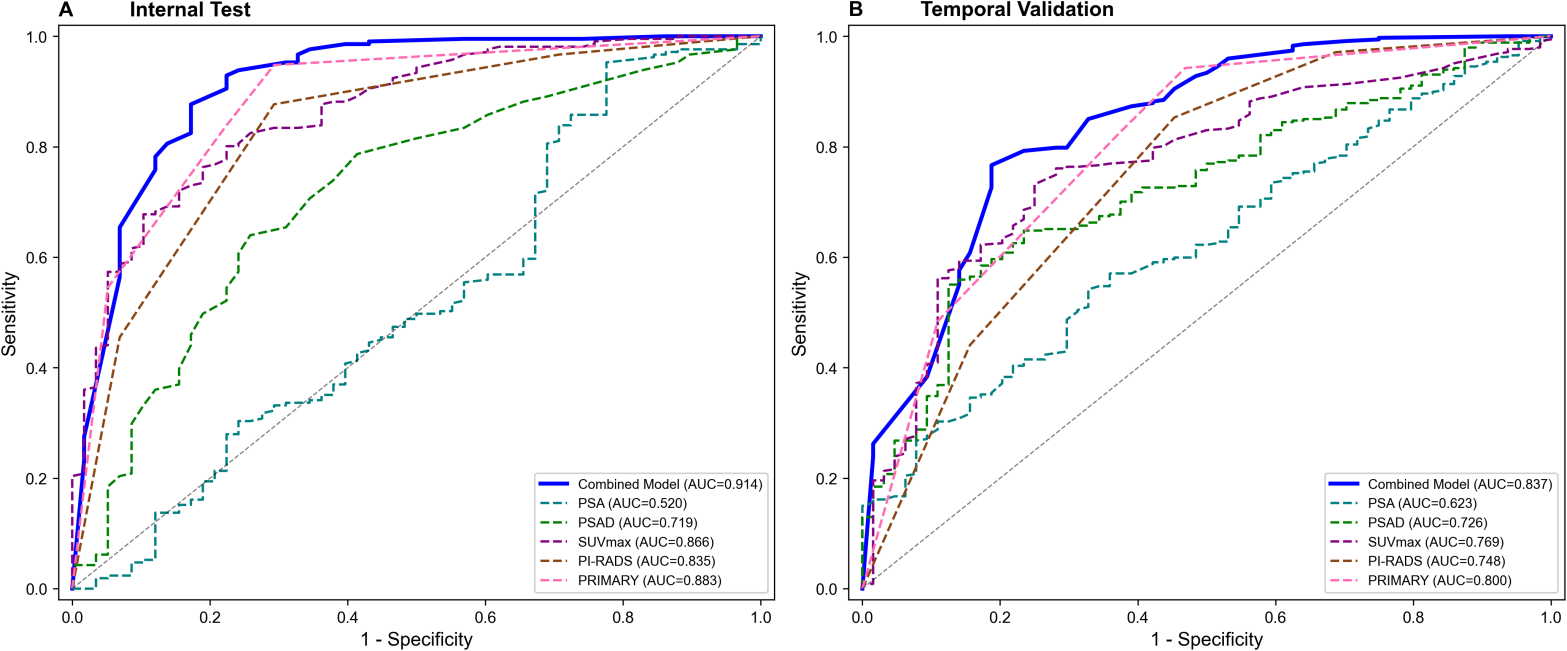
ROC curves for predicting csPCa. **(A)** ROC Curves of Internal Test. **(B)** ROC Curves of Temporal Validation. PSA, prostate specific antigen; PSAD, prostate specific antigen density; SUVmax, maximum standardized uptake value; PI-RADS, Prostate Imaging Reporting and Data System.

**Figure 7:**
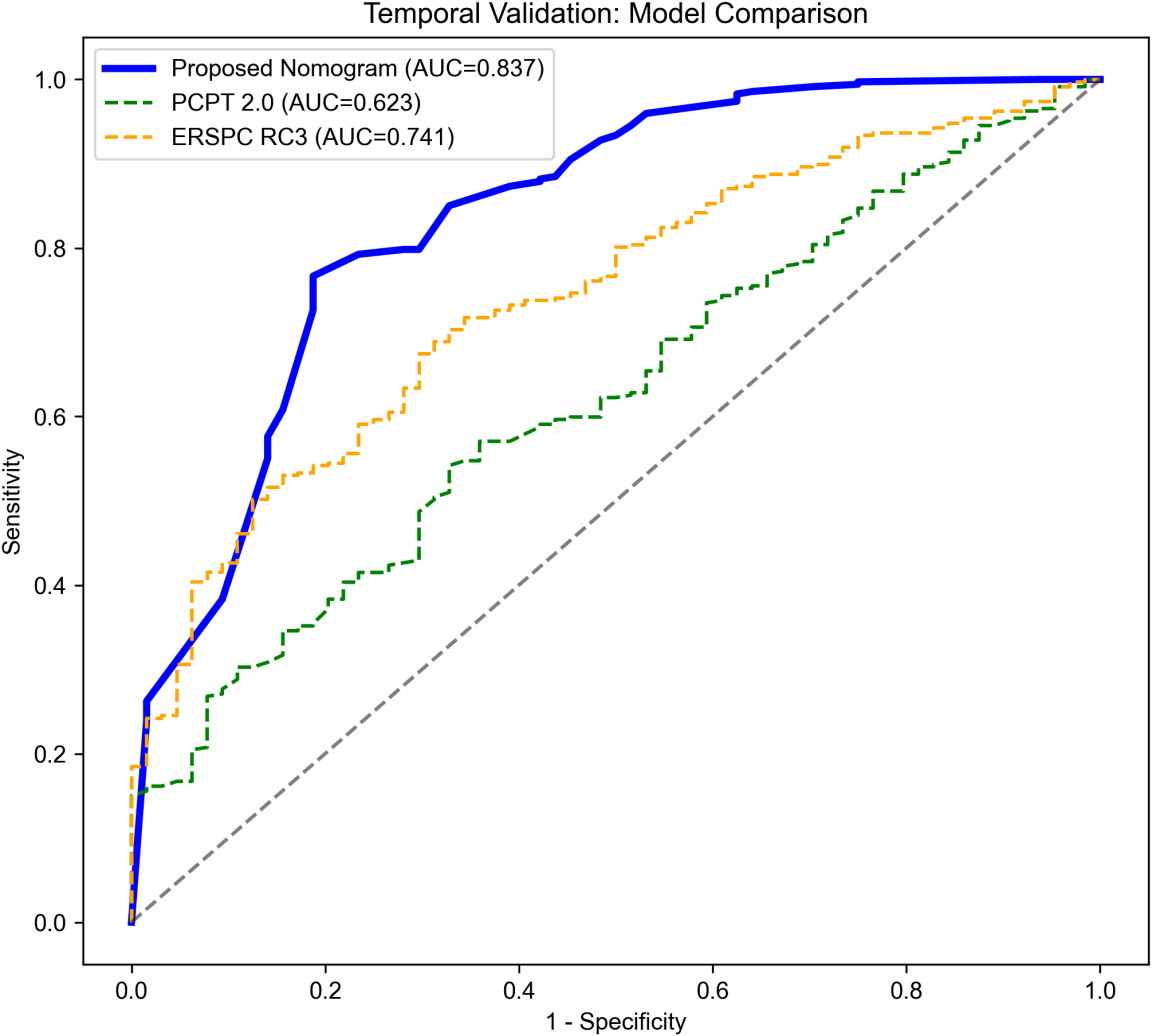
Head-to-head comparison of receiver operating characteristic (ROC) curves in the temporal validation cohort.

**Figure 8:**
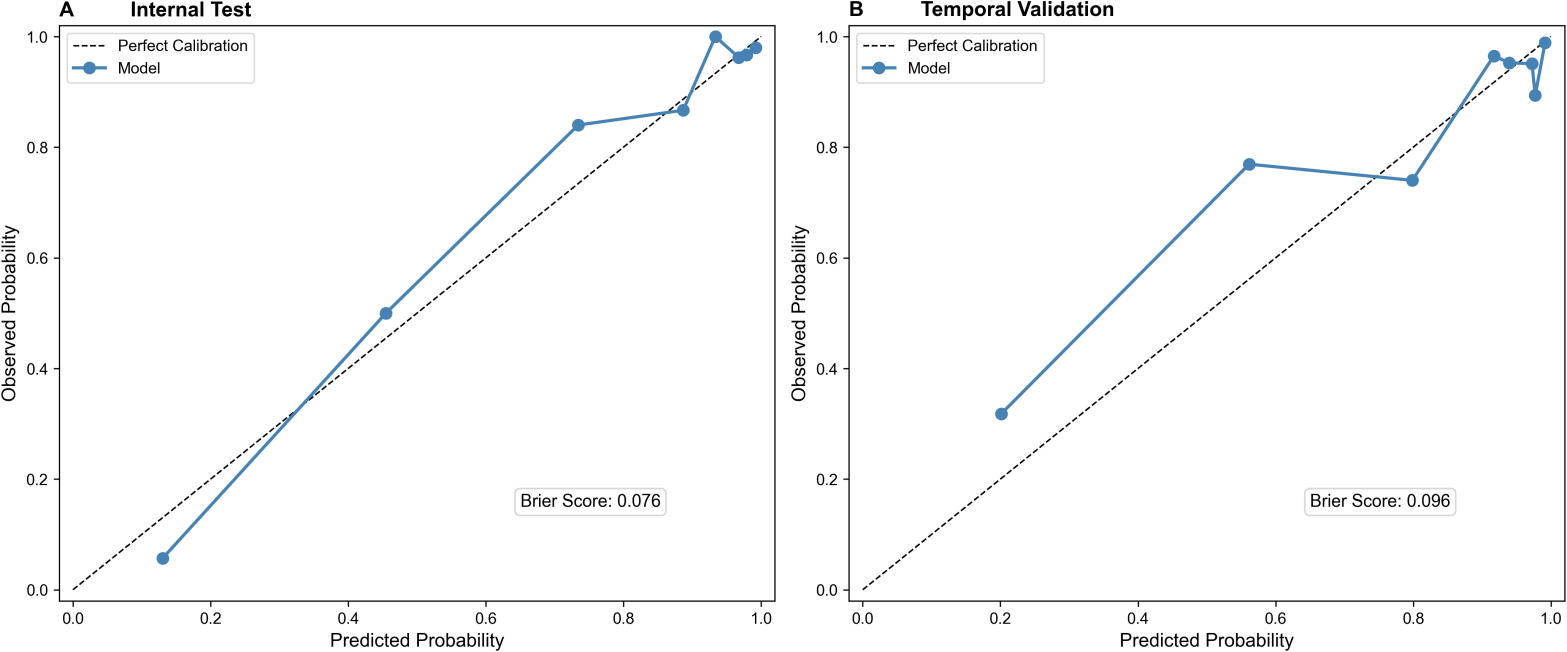
Calibration curves for predicting csPCa. **(A)** Calibration curve of Internal Test. **(B)** Calibration curve of Temporal Validation.

To rigorously evaluate potential overfitting, internal validation using 1000 bootstrap resamples was performed. The apparent AUC was 0.9156 with a low calculated optimism of 0.0026, yielding an optimism-corrected AUC of 0.9130. In the internal test set, the model achieved an AUC of 0.914 (95% CI: 0.882-0.941) (**Figure 6A**). In the temporally separated temporal validation cohort, the model maintained an AUC of 0.837 (95% CI: 0.778-0.891) (**Figure 6B**). Furthermore, a head-to-head comparison in the temporal validation cohort demonstrated that our proposed model (AUC 0.837) possessed superior discriminative ability compared to the established PCPT 2.0 (AUC 0.623) and ERSPC RC3 (AUC 0.741) risk calculators (**Figure 7**). The calibration plots demonstrated good agreement between predicted probabilities and observed outcomes in both the internal test set and temporal validation cohort (**Figure 8**), with Brier scores of 0.076 and 0.096, respectively.

**Figure 9:**
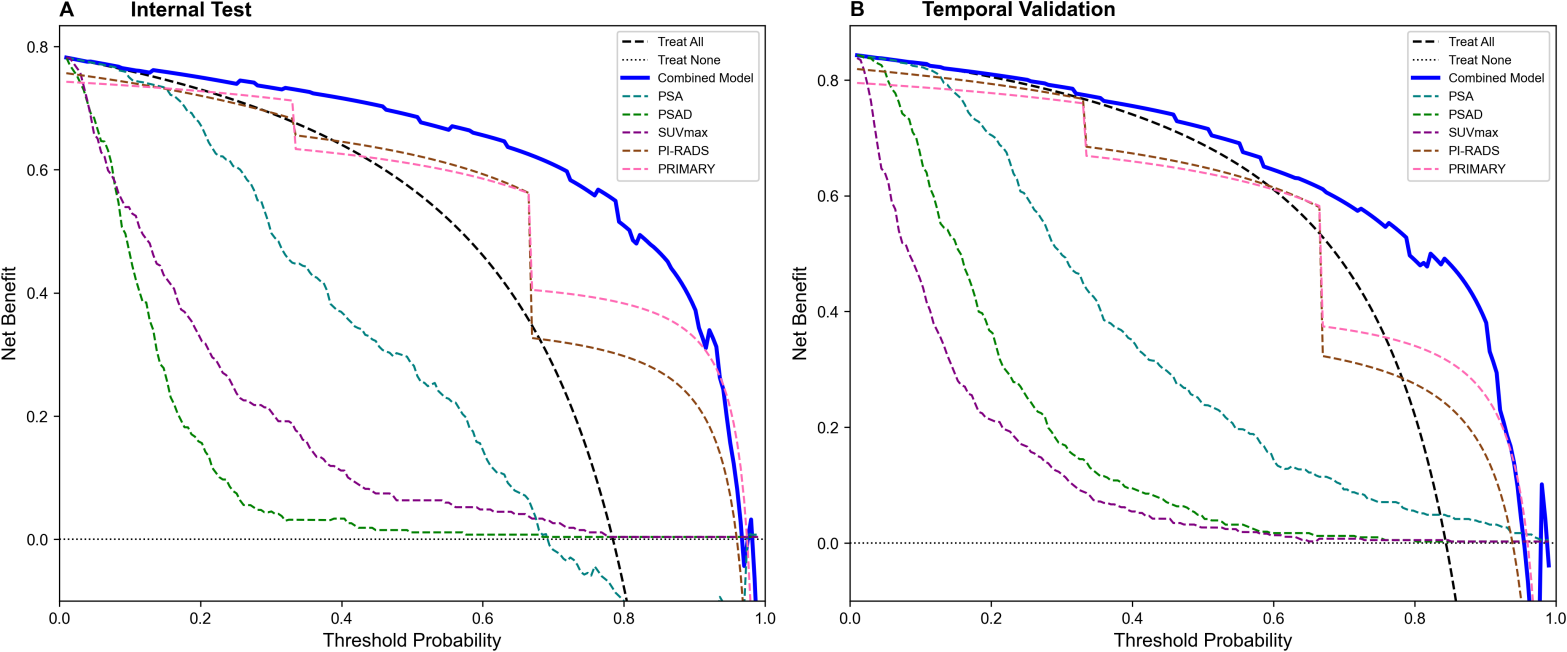
Decision curve analysis (DCA) for predicting csPCa. **(A)** Net benefit curves of Internal Test Set. **(B)** Net benefit curves of Temporal Validation Set. PSA, prostate specific antigen; PSAD, prostate specific antigen density; SUVmax, maximum standardized uptake value; PI-RADS, Prostate Imaging Reporting and Data System.

Decision curve analysis showed that the combined model provided superior net benefit across a wide range of threshold probabilities (20% to 90%) in both internal and external cohorts (**Figure 9**), supporting its robust clinical utility.

## Discussion

We developed and validated a multivariable prediction model for csPCa integrating clinical variables, mpMRI parameters, and 18F-PSMA PET/CT findings. In the training set, the model achieved an apparent AUC of 0.916. It demonstrated excellent discriminative ability in the internal test set (AUC 0.914, 95% CI: 0.882-0.941) and maintained robust transportability in the temporal validation cohort (AUC 0.837, 95% CI: 0.778-0.891).

While mpMRI represents the current standard for pre-biopsy risk stratification, it is primarily limited by moderate interobserver variability and suboptimal specificity. The integration of 18F-PSMA PET/CT provides complementary molecular insight, specifically mapping the overexpression of prostate-specific membrane antigen in clinically significant lesions (21). Recent evidence, such as the PRIMARY2 trial, highlights the critical role of PSMA PET strategies in safely reducing unnecessary biopsies. Furthermore, there is an evolving clinical landscape toward utilizing quantitative PET parameters, such as SUVmax, for robust risk stratification and direct therapeutic decision-making (22). Our integrated approach further demonstrates that combining PSMA PET/CT, mpMRI, and PSAD optimizes risk stratification beyond what either modality can achieve alone. In our rigorous dual-stage variable screening, PRIMARY score demonstrated profound independent prognostic value, whereas quantitative SUVmax and MRI-based T-stage were eliminated during multivariable stepwise adjustment. This exclusion underscores a critical principle of statistical parsimony: the variance explained by SUVmax and T-stage was highly collinear with the combined qualitative assessment provided by PI-RADS and PRIMARY scores. Consequently, our 3-variable model avoids the redundancy of including overlapping imaging metrics, offering a streamlined yet powerful tool for clinical decision-making. It is worth noting that, based on our nomogram, the isolated predictive probability of a PRIMARY score of 5 appears somewhat lower than the >90% detection rate reported in the original PRIMARY publication (13). This apparent reduction is an inherent characteristic of our multivariable model design. Because the overall predictive weight is systematically distributed across three distinct clinical and imaging variables (PRIMARY score, PI-RADS score, and PSAD), the maximum predictive threshold can only be reached when these complementary parameters are combined. Therefore, a high PRIMARY score in our model should not be interpreted in isolation, but rather as a synergistic component of the integrated approach, which ultimately yields a more comprehensive risk stratification.

Furthermore, our final model strictly retained prostate-specific antigen density (PSAD) as the sole clinical biomarker, actively excluding raw serum PSA and prostate volume to circumvent computational collinearity. PSAD has been extensively validated in contemporary literature as a superior metric to PSA, effectively neutralizing the confounding effect of benign prostatic hyperplasia and significantly improving diagnostic specificity for csPCa (9, 23). Our stepwise AIC selection objectively confirmed that PSAD provides optimal, non-redundant predictive weight alongside advanced imaging parameters.

To maximize clinical applicability, we evaluated our nomogram across two distinct probability thresholds. The Youden Index identified an optimal cutoff of ≥84%, which balances sensitivity and specificity for confident csPCa diagnosis. Conversely, selecting a lower threshold of ≥46% drastically elevates sensitivity to 96.0%. This lower cutoff is clinically recommended for screening purposes, aligning with the primary objective of pre-biopsy risk stratification: safely avoiding unnecessary biopsies without missing high-risk cancers, analogous to the triage thresholds employed by contemporary risk calculators (24).

The observed reduction in AUC from 0.914 in the internal test set to 0.837 in the temporal validation cohort represents a natural calibration drift commonly encountered when prediction models are strictly transported to temporally or geographically distinct populations (17, 20). Contributing factors likely include differences in baseline cohort characteristics—such as variations in csPCa prevalence and median PSAD—as well as evolving referral patterns over time. Additionally, this study has limitations, including its retrospective nature which may introduce selection bias, and the relatively small sample size in the validation cohort. Future prospective multicenter trials are warranted to further refine these thresholds and establish broad generalizability.

Currently, several pre-biopsy risk calculators are utilized in clinical practice to assist decision-making. For instance, the Prostate Cancer Prevention Trial (PCPT) risk calculator, incorporating clinical variables such as age and PSA, yields an area under the curve (AUC) of approximately 0.70 to 0.75 (25). Similarly, the European Randomized Study of Screening for Prostate Cancer (ERSPC) risk calculator demonstrates an AUC of 0.75 to 0.80 (26). More advanced tools, such as the Stockholm3 model, which integrates clinical variables with protein markers and genetic polymorphisms, can achieve AUCs of 0.80 to 0.85, albeit at a significantly higher diagnostic cost (27). Compared to these existing risk models, a key innovation of our study is the formal integration of quantitative and qualitative PSMA PET parameters alongside standard clinical and MRI factors, ultimately yielding robust diagnostic discrimination (temporal validation AUC 0.837) without the requisite for complex genetic profiling.

Several limitations should be acknowledged. First, despite consecutive enrollment, the retrospective component may be subject to selection bias. Second, the reference standard was systematic biopsy rather than whole-mount prostatectomy pathology, which may underestimate true tumor burden (28). Third, the prevalence of csPCa in our cohort is remarkably high (78.3% and 84.4%). This reflects a tertiary-center spectrum bias, as our population primarily consists of patients referred with highly elevated PSA levels or suspicious prior imaging. This bias may overestimate the positive predictive value and limits the direct applicability of our model to primary screening populations; the nomogram is primarily intended to aid decision-making in high-risk populations where multimodal imaging is already being considered. Fourth, MRI-based T staging rather than DRE-based clinical T staging was used (6). Fifth, despite temporal validation, further validation in larger, multi-centric, and prospective settings is necessary prior to wide-scale clinical adoption. Furthermore, the combined utilization of mpMRI and 18F-PSMA PET/CT imposes a significant financial burden and relies on advanced medical infrastructure. We acknowledge that this integrated approach may be cost-prohibitive and less accessible in developing countries or resource-limited settings. Consequently, while our model demonstrates excellent theoretical diagnostic performance, its immediate practical application may be restricted to well-resourced tertiary centers rather than routine primary screening. Finally, while all scans were reviewed by highly experienced readers, we did not formally calculate inter-observer agreement metrics (such as Cohen’s Kappa) at the time of study design, which represents a potential source of bias (29).

Future studies should focus on prospective multicenter validation, evaluation in populations with lower csPCa prevalence, and incorporation of emerging biomarkers. The model may serve as a framework for integrating multimodal imaging data in prostate cancer diagnosis (30).

## Conclusion

We developed and validated a multivariable prediction model for csPCa integrating clinical variables, mpMRI parameters, and 18F-PSMA PET/CT findings. Compared to existing risk models, a key innovation of our study is the formal integration of quantitative and qualitative PSMA PET parameters alongside standard clinical and MRI factors. The model achieved an AUC of 0.916 in the training set, with maintained discriminative ability in both internal test (AUC = 0.914) and temporal validation (AUC = 0.837) cohorts.

## Data Availability

The raw data supporting the conclusions of this article will be made available by the authors, without undue reservation.
