# Supplementary material for: A multivariable prediction model combining 18F-PSMA PET/CT and mpMRI for clinically significant prostate cancer: development and validation
Source: Front Oncol. 2026 May 18;16:1835850. doi: 10.3389/fonc.2026.1835850 (PMC13223179; doi:10.3389/fonc.2026.1835850)
Supplement: Supplementary file 1 [file Table1.docx]

**Supplementary Material**

**Supplementary Table S1.** PRIMARY Scoring System for Intraprostatic PSMA Uptake

The PRIMARY scoring system was developed by Emmett et al. (1) to standardize the qualitative assessment of intraprostatic PSMA uptake on PET/CT imaging using a combination of pattern information and SUVmax:

| **Score** | **Pattern Description** | **Criteria** |
| --- | --- | --- |
| 1 | No pattern, low-grade activity | No focal uptake above background |
| 2 | Diffuse TZ or symmetric CZ activity | Diffuse transition zone or symmetric central zone activity without focal uptake |
| 3 | Focal TZ activity | Focal transition zone activity visually greater than twice background TZ activity |
| 4 | Focal PZ activity | Focal peripheral zone activity |
| 5 | Any pattern with high SUVmax | Any pattern with SUVmax ≥12 |

*Abbreviations: TZ, transition zone; CZ, central zone; PZ, peripheral zone; SUVmax, maximum standardized uptake value.*

*Note: In cases with multiple patterns, the PRIMARY score represents the most clinically significant pattern (focal pattern above diffuse or symmetric, PZ above TZ, and SUVmax ≥12 above any reported pattern).*

Reference: (1) Emmett L, Buber N, Cusick T, et al. The PRIMARY Score: Using Intraprostatic 68Ga-PSMA PET/CT Patterns. J Nucl Med. 2022;63(11):1644-1650.
